# Innovative Insights on the Thin Square Plate Large Deflection Problem

**DOI:** 10.3390/ma16216967

**Published:** 2023-10-30

**Authors:** Gilad Hakim, Haim Abramovich

**Affiliations:** Technion Faculty of Aerospace Engineering, Israel Institute of Technology, I.I.T., Haifa 32000, Israel; ghakim@outlook.com

**Keywords:** square thin plate, large deflection, von Kármán equations, membrane stress, non-linear load–deflection curve, finite element analysis, Fourier series, simply supported movable edges

## Abstract

Thin plates subjected to transverse load and undergoing large deflections have been widely studied and published in the literature. However, there is still a lack of information and understanding about the membrane stresses created under large deflections and their associated Airy stress function, as displayed in the well-known von Kármán equations set. The present study aims at providing explicit expressions for the membrane stresses, the deflections, and the Airy stress function for a general square plate area vertically uniformly loaded to reach large deflection state. This was obtained by using the results of a high-fidelity finite element analysis applied on a lateral loaded simply supported thin square plate, which are then casted to yield approximate Fourier series expressions for the membrane stresses, deflections, and the Airy stress function. The stress map figures provide a good understanding of the critical points on the plate, while the explicit mathematical expressions enabled the calculation of deflections and stresses for the entire plate area. Among other interesting findings, the presence of relatively high tensile and compressive membrane stresses existing near the plate edges was revealed, which might lead to potential failure hazards. The derivatives of the deflections and the Airy stress function enabled the validation of the large deflections von Kármán equations set for the investigated case, and it turned out that the generated expressions for the stresses and the lateral deflection based on a high-fidelity finite element model satisfy the second equation with a good accuracy, while the first one remains to further be investigated. Moreover, using the generated explicit equations, the load influence on the deflections and stresses was also analyzed to yield general novel expressions for the medium and very large deflections states. To generalize the investigated case, the stresses and the deflections were non-dimensionalized so they can be used for any material and plate dimensions.

## 1. Introduction

The behavior of flat plates subjected to various loads has attracted over the years great attention due to its technological importance. This immense problem is subdivided into many sections. It can be divided by load type, thickness, perimeter shape, material properties, small vs. large deflection, shear deformability, and more. Among these, the problem of a thin isotropic square plate loaded by transversal pressure is considered as a classical problem, deeply investigated in the literature.

Small deflections of a plate, not exceeding a fraction of the plate thickness, present a linear load–deflection behavior with good and satisfactory solutions being available in the literature. However, large deflections of a plate in the range of a few times the thickness and higher have raised severe difficulties to reach solutions. The load–deflection graph line is non-linear, suggesting an additional mechanism that accumulates elastic energy, in addition to the bending energy accumulation due to the applied loading.

A major breakthrough for the large deflection problem was made by Theodore von Kármán in 1910 [1]. He published a set of two differential equations that describes the plate’s large deflection behavior, considering the in-plane deformations, stresses, and the elastic energy. Unfortunately, the von-Kármán equations set is very complicated for solving. To date, there is still no closed-form analytic non-trivial solution that satisfies both the equations and the boundary conditions for rectangular plates. Nevertheless, many approximated and numerical solutions were published. The approximate methods suggest solutions, however with severe limitations. Most of them are not easy to use, and the non-linear nature of the plate hardening effect in large deflection is not evident (see, for example, [2,3,4,5,6,7,8,9,10,11,12,13,14,15,16,17,18,19,20,21,22,23,24,25,26,27,28]). Although these references were widely referred to and described in [29], for the convenience of readers, a detailed literature survey for Refs. [2,3,4,5,6,7,8,9,10,11,12,13,14,15,16,17,18,19,20,21,22,23,24,25,26,27,28] is presented in Appendix D.

The present study is confined to the analysis of thin square plates loaded by transverse pressure with simply supported edges. In addition, the edges are allowed to freely move within the plate plane—to be named “movable edges”. The deflections are considered moderately large, in which the in-plane stresses create a hardening effect, making the plate more rigid while the load increases. The term “moderately” stands for a plate having large deflections with relatively small rotations.

Despite the extensive research conducted in this field, there is still a lack of explicit mathematical expressions and graphical pictures presenting the deflections and the in-plane stresses for the entire plate due to normal loading. Moreover, no appropriate equation for the Airy stress function (ASF) that relates to the in-plane stress field is available in the literature. Therefore, the present study aims at filling up this knowledge gap.

Under a specified load, the plate would deform. The general shape of the deformed plate is rather intuitive and easy to predict. Nevertheless, the exact function of the deformed plate surface is still difficult to cast. Unlike deflections, the membrane stress field created within the loaded plate during large deflections is beyond our natural perception. This is a real problem when trying to find the resulting stress, the critical points, and the corresponding ASF.

The purpose of the present study is to formulate, generate, and present high-fidelity approximated mathematical expressions and graphical pictures of the deflections, tensile and shear in-plane stresses, and ASF and its derivatives for the entire plate area. Moreover, using the generated explicit equations, the load influence on the deflections and stresses was also analyzed to yield general novel expressions for the medium and very large deflections states.

The various expressions were generated based on a high-fidelity finite element (FE) model developed and validated in Hakim & Abramovich [29], which presented its results, but without mathematical expressions, which are presented in the present article. The newly generated expressions would enable the use of an Excel worksheet to easily calculate and display these parameters at any point on the plate, and for any applied distributed load level, without elaborative FE models, thus yielding a powerful practical application to be used by engineers and scholars, stemming from the present advanced numerical study.

Once the derivatives of the ASF and the deflection are numerically known, the validity of the von Kármán equations set at any point on the plate was evaluated, yielding a good accuracy.

During the large deflection plate analysis, a surprising finding was evident, namely the existence of strong in-plane tensile and compressive stresses within the loaded plate. The compressive stresses have the potential to create local buckling, which might be considered as a failure. The strong tensile stresses might create cracks and breaking failures. These potential failure sources have been rarely noticed and published in the past in the open literature.

## 2. Materials and Methods

### 2.1. Problem Definition

The square isotropic thin plate problem discussed in the present study has the following variables: the width and the length are each *a*, the plate’s thickness is *h*, the material Young’s modulus is *E*, and the Poisson’s ratio is *ν*.

**Figure 1:**
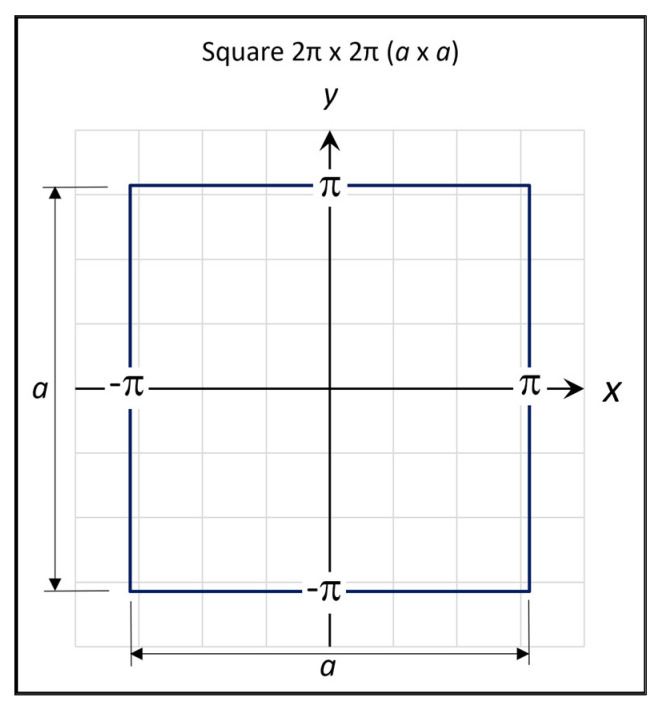
The defined square plate.

In order to ease the calculation procedure, the horizontal and the vertical axes are defined to obtain a square 2π × 2π plate. The origin (0, 0) of the *x*, *y* system is located at the plate’s mid-point, as shown in Figure 1.

The plate displayed in Figure 1 is assumed to be transversely loaded with a uniformly distributed pressure $q\left(x,y\right)=q$, resulting in deflections, $w\left(x,y\right)$, in the *z* direction as well as in-plane displacements.

For the case considered in the present study, the plate edges are simply supported, i.e., *w* = 0 at the edges, while the plate at the edge is free to rotate around an axis, which is the edge itself. In contrast, the holding support frame keeps the plate edge straight, preventing any curvature along the edge. Consequentially, at the edges, there are no bending moments in both *x* and *y* directions. These boundary conditions (BC) are designated as SSSS for the four edges satisfying the simply supported condition, i.e., in all around edges transverse deflection and bending moment are zero.

Additionally, the plate edges are free to move in the *x*, *y* plane within the support frame, resulting in zero internal in-plane membrane forces and stress at the edges, perpendicular to the edge. In addition, since there are no in-plane external forces on the plate’s edges, including forces parallel to the edge, the shear membrane forces and the shear stresses at the plate edges are zero as well. This arrangement is named “movable edges”, which is different from the more common case where the plate edges are firmly held: “immovable edges”.

Therefore, the BC used for the present case are given as:

(1) $$w=0,\tau_{xy}=0,\;M_{x}=M_{y}=0\;\mathrm{at}\;\mathrm{all}\;\mathrm{four}\;\mathrm{edges}$$

(2) $$\sigma_{xx}=0\;\mathrm{at}\;x=\pm \pi ,\;\sigma_{yy}=0\;\mathrm{at}\;y=\pm \pi$$

where *w* is the lateral deflection, $\tau_{xy}$ is the shear stress, and $M_{x}$ and $M_{y}$ are the bending moments. One should note that the stress $\sigma_{xx}$ does not vanish at y=±π and $\sigma_{yy}$ does not vanish at x=±π. This means that at the edges, there are tensile/compressive in-plane stresses acting in the direction parallel to the edge. These stresses are the result of the plate’s large deformation, not a result of external applied forces.

In mathematical terms, the boundary conditions can be written as:

(3) $$w\left(\pm \pi ,\pm \pi \right)=0,\;{\left.\frac{\partial^{2}w}{\partial y^{2}}\right|}_{x,y=\pm \pi }={\left.\frac{\partial^{2}w}{\partial x^{2}}\right|}_{x,y=\pm \pi }=0$$

and the in-plane stresses perpendicular to the edges are zero, namely

(4) $${\left.\sigma_{xx}\right|}_{x=\pm \pi }=0,\;{\left.\sigma_{yy}\right|}_{y=\pm \pi }=0$$

In addition, no in-plane shear forces and stresses exist between the support frame and the plate edges, meaning that

(5) $${\left.\sigma_{xy}\right|}_{x,y=\pm \pi }=0$$

One should remember that when the plate deflections due to the transversal load are small in relation to the plate thickness, the plate presents a linear load–deflection behavior, described by the classical plate theory (CPT). This linear case has several closed-form solutions shown in many previously published sources, see Hakim & Abramovich [30] for more details. However, for the moderated large deflections case, a general closed-form solution is still not available, and the use of von Kármán equations is usually advised. These equations have the following form, as presented in Timoshenko [31] (p. 417):

(6) $$\frac{\partial^{4}\varphi }{\partial x^{4}}+2\frac{\partial^{4}\varphi }{\partial x^{2}\partial y^{2}}+\frac{\partial^{4}\varphi }{\partial y^{4}}=E\left({\left(\frac{\partial^{2}w}{\partial x\partial y}\right)}^{2}-\frac{\partial^{2}w}{\partial x^{2}}\frac{\partial^{2}w}{\partial y^{2}}\right)$$

(7) $$D\left(\frac{\partial^{4}w}{\partial x^{4}}+2\frac{\partial^{4}w}{\partial x^{2}\partial y^{2}}+\frac{\partial^{4}w}{\partial y^{4}}\right)=q\left(x,y\right)+h\left(\frac{\partial^{2}\varphi }{\partial y^{2}}\frac{\partial^{2}w}{\partial x^{2}}+\frac{\partial^{2}\varphi }{\partial x^{2}}\frac{\partial^{2}w}{\partial y^{2}}-2\frac{\partial^{2}\varphi }{\partial x\partial y}\frac{\partial^{2}w}{\partial x\partial y}\right)$$

where $\varphi \left(x,y\right)$ is the Airy stress function (ASF), $w\left(x,y\right)$ is the lateral deflection, $q\left(x,y\right)$ is the applied distributed load, *E* is the extensional elasticity modulus, *h* is the plate thickness, *ν* is the Poisson’s ratio, and *D* is the plate flexural stiffness:

$$D=\frac{Eh^{3}}{12\left(1-\nu^{2}\right)}$$

As presented above, the load is a uniformly distributed pressure: $q\left(x,y\right)=q$.

It is interesting to note, as pointed out by Bakker et al. [32], that Equations (6) and (7) are a simplification of Marguerre’s [33] equations for plates having initial imperfections and subjected to in-plane and transverse loads (the initial imperfection is taken as zero in Equations (6) and (7)).

Notice that the ASF is an unknown two-dimensional (2D) scalar function designated $\varphi \left(x,y\right)$ with the relations to the plate in-plane membrane stresses:

(8) $$\sigma_{xx}=\frac{\partial^{2}\varphi }{\partial y^{2}}\;,\;\;\;\sigma_{yy}=\frac{\partial^{2}\varphi }{\partial x^{2}}\;,\;\;\;\tau_{xy}=-\frac{\partial^{2}\varphi }{\partial x\partial y}$$

For linear systems, and in the absence of body forces, thermal gradients, and potential fields, the ASF must satisfy the 2D bi-harmonic equation:

(9) $$\nabla^{4}\varphi =\frac{\partial^{4}\varphi }{\partial x^{4}}+2\frac{\partial^{4}\varphi }{\partial^{2}x\partial^{2}y}+\frac{\partial^{4}\varphi }{\partial y^{4}}=0$$

For non-linear systems, however, this bi-harmonic equation requirement does not exist. Therefore, the left-hand side of the first equation of the von Kármán equations set in Equation (6) does not automatically vanish and therefore must be considered.

Another important restriction on the ASF is that it must satisfy the stress BC Equations (4) and (5), namely

(10) $${\left.\frac{\partial^{2}\varphi }{\partial y^{2}}\right|}_{x=\pm \pi }=0,\;\;\;{\left.\frac{\partial^{2}\varphi }{\partial x^{2}}\right|}_{y=\pm \pi }=0,\;\;\;\;{\left.\frac{\partial^{2}\varphi }{\partial x\partial y}\right|}_{x,y=\pm \pi }=0$$

### 2.2. Preliminary Assumptions

There are several assumptions that are the basis for the formulation of the von Kármán equation set. For example, squares and products of certain in-plane displacement derivatives are considered small and therefore negligible, see, for example, Bhaskar [34] p. 306 (2013). By using the equations set, we implicitly accept these assumptions.

Another preliminary assumption is that both the deflection and the ASF are a multiplication of a shape function (*SF*) by a load function (*LF*), namely:

(11) $$w\left(x,y,q\right)=SF1_{d}\left(x,y\right)\cdot LF1\left(q\right)$$

where $SF1_{d}$ is the shape function and LF1 is the load function, both for the deflection expression.

(12) $$\varphi \left(x,y,q\right)=SF2\left(x,y\right)\cdot {LF2}_{\varphi }\left(q\right)$$

where SF2 is the shape function and ${LF2}_{\varphi }$ is the load function, both for the ASF expression.

This means that the general shape of the functions remains the same, while the load is changing.

From (12), it follows that both shear and tensile membrane stress functions are also a shape–load multiplication.

The purpose of function indices 1,2 is to distinguish between the deflection function (1) and stress target functions (2). This assumption will be utilized later in Section 3.4.

The last preliminary assumption is that the load *q* is assumed to be a polynomial function of the plate mid-point deflection $w_{0}$, namely

(13) $$q=K_{1}w_{0}+K_{3}{w_{0}}^{3}$$

with *K*_1_ and *K*_3_ being constants, as presented in Hakim [30].

The above assumption is the result of many existing solutions mentioned in [30], laboratory loading tests done on various plates, and a few non-linear finite element analyses (FEA) performed (see Hakim & Abramovich [29], Siemens [35]).

The motivation for applying this assumption stems from its popularity among many researchers. Note, however, that this assumption will be later rechecked during the present study for its range of validity and eventually be modified.

### 2.3. Finite Element Analysis (FEA) of the Squared Plate

An FEA of the squared plate shown in Figure 1 was then performed. The analysis code was Femap 2021.1 from Siemens, with Simcenter Nastran [35] as the code processor. A 6.28 m by 6.28 m plate of 12 mm thickness made of an isotropic material (*E* = 2.4 GPa, Poisson’s Ratio *ν* = 0.38) with appropriate BC was transversely loaded by an 800 Pa uniformly distributed pressure. A non-linear static analysis was performed for 10,000 (100 × 100) quad plate-type elements with a 6.28 cm element size. The non-linear code increased the load in 20 steps, while in each step the deflections and the stresses were recalculated and used as a starting point for the next step. Each of these steps had internal iterations to verify its convergence. Upon completion of the run, all final deflections and membrane forces of the entire plate were transferred to an Excel sheet. Note that the deflection and membrane force data were stored in an Excel 103 × 103 data table with the following modifications, needed to correctly perform the various follow up calculations:

- Deflections at the center of element were calculated as the average of the four elements’ corner deflections, while the edges’ deflection values were set to 0.
- The *X*-direction membrane force was modified to *X* tensile stress $\sigma_{xx}\left(x,y\right)$ and at the relevant edges, the values were set to 0.
- The shear membrane force was modified to *XY* shear stress $\tau_{xy}\left(x,y\right)$ and at the edges and on *X* and *Y* axes, its values were set to 0.

Graphical calculation pictures of the FEA results were also saved for further processing.

The isotropic material used in the analysis is polycarbonate (PC), a tough transparent polymer used in many technical applications such as aircraft cockpit canopies, safety goggles, compact disks, and greenhouse glazing.

## 3. Results

### 3.1. Generation of Mathematical Expressions for Deflections, Stresses, and ASF

Numerical partial derivatives of both the deflection and the shear stresses are calculated using the finite difference schemes presented in Appendix A. The central difference scheme is used for most cells, while forward and backward schemes are used for the edges.

After two subsequent numerical derivations (using the generated Excel worksheet, see Section 2.3 above) in the *x* direction, the second derivative $\frac{\partial^{2}w}{\partial x^{2}}$ is obtained. Note that the *y*-direction second derivative, $\frac{\partial^{2}w}{\partial y^{2}}$, has a similar appearance but with a 90° rotation in the *xy* plane (see also Appendix B).

**Figure 2:**
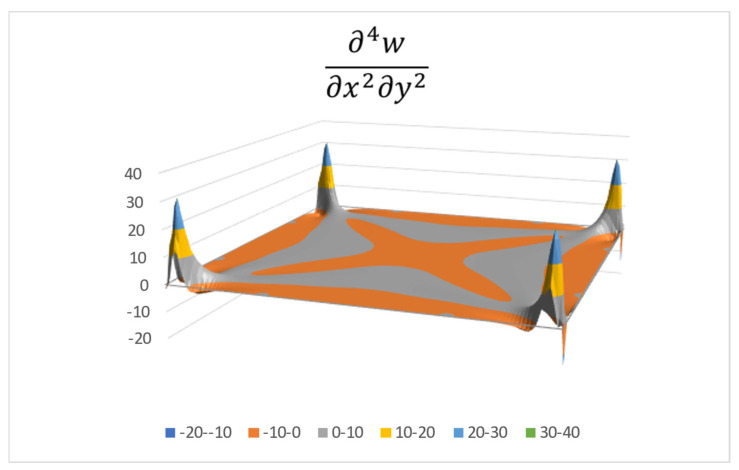
Fourth mixed derivative plate deflection.

Then, after another two subsequent derivations in the *y* direction, the fourth mixed derivative, $\frac{\partial^{4}w}{\partial x^{2}\partial y^{2}}$, is generated. One should note that numerical “noise” ripples begin to be evident on the surface in the vicinity of the plate’s corners (see a typical case in Figure 2). This is a known effect of successive numerical derivations, and in a real plate case it does not exist.

The various colors in Figure 2 (and in the following figures) represent the function values, according to the legend at the bottom of the figure.

Finally, the *x*-direction fourth derivative, $\frac{\partial^{4}w}{\partial x^{4}}$, is then generated using the process described in Appendix B. Note that the *y*-direction fourth derivative, $\frac{\partial^{4}w}{\partial y^{4}}$, has a similar appearance but with a 90° rotation in the *xy* plane.

A Fourier series is then matched to the deflection distribution using the fourth mixed derivative, $\frac{\partial^{4}w}{\partial x^{2}\partial y^{2}}$, as described in Appendix B. As can be seen in Figure 2, this function is symmetric about both *x* and *y* axes, so a cosine-cosine series is suitable to be used, namely

(14) $$\frac{\partial^{4}w}{\partial x^{2}\partial y^{2}}=\sum_{m=0}^{\infty }\sum_{n=0}^{\infty }C_{mn}\;\cos\;mx\;\cos\;ny$$

The $C_{mn}$ Fourier series coefficients are then found using the method presented in Appendix B. The calculated 19 × 19 = 361 (a total of 190 independent) coefficients are presented in Appendix C.

**Figure 3:**
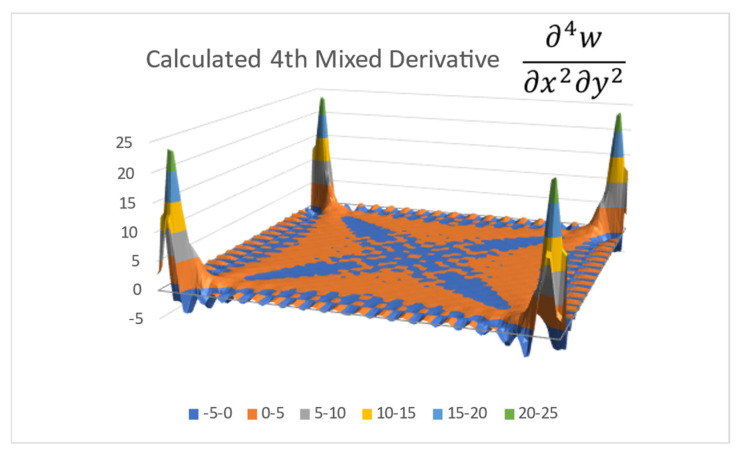
Fourier series-calculated fourth mixed derivative.

The resulted approximated Fourier series function with its $C_{mn}$ coefficients is graphically displayed in Figure 3, well resembling the distribution presented in Figure 2.

According to Appendix B, the obtained Fourier series can now be integrated twice in the *x* direction and then twice in the *y* direction to yield the approximated deflection function. However, before doing so, one has to separate the series for the indices *m* = 0 and *n* = 0 to avoid division by zero, namely

(15) $$\frac{\partial^{4}w}{\partial x^{2}\partial y^{2}}\approx \sum_{m=0}^{18}\sum_{n=0}^{18}C_{mn}\cos\;mx\;\cos ny=C_{0,0}+\sum_{n=1}^{18}C_{0,n}\cos\;ny+\sum_{m=1}^{18}C_{m,0}\cos\;mx+\sum_{m=1}^{18}\sum_{n=1}^{18}C_{mn}\cos\;mx\;ny$$

Integrating twice in the *x* direction and twice in the *y* direction yields

(16) $$\begin{array}{c} w\left(x,y\right)\approx x^{2}y^{2}C_{0,0}-\frac{1}{2}x^{2}\sum_{n=1}^{18}\frac{C_{0,n}}{n^{2}}\cos\;ny-\frac{1}{2}y^{2}\sum_{m=1}^{18}\frac{C_{m,0}}{m^{2}}\cos\;mx+\sum_{m=1}^{18}\sum_{n=1}^{18}\frac{C_{mn}}{m^{2}n^{2}}\cos\;mx\cos\;ny\\ +x\cdot F_{1}\left(y\right)+F_{2}\left(y\right)+y\cdot G_{1}\left(x\right)+G_{2}\left(x\right) \end{array}$$

where $F_{1}$, $F_{2}$, $G_{1}$, and $G_{2}$ are arbitrary integration functions to be found according to the case solved.

Looking at the terms of the expression presented in Equation (16) and comparing them with the FEA lateral deflection $w\left(x,y\right)$, it is obvious that $F_{1}\left(y\right)=G_{1}\left(x\right)=0$ since the deflection must be symmetric about both *x* and *y* axes.

The symmetry also causes $G_{2}\left(x\right)$ and $F_{2}\left(y\right)$ to have the same form, with a symmetry about its axes. These functions can be approximated by a cosine Fourier series, for which a finite number of coefficients $A_{j}$ can be found by fitting the deflection function Equation (16) to the FEA-generated deflection, while the terms with $C_{mn}$ are already known. In our case, 50 $A_{j}$ coefficients (0–49) were found, yielding

(17) $$G_{2}\left(x\right)=\sum_{j=0}^{49}A_{j}\cos jx\;,\;\;\;F_{2}\left(y\right)=\sum_{j=0}^{49}A_{j}\cos\;jy\;\;\to \;\;G_{2}\left(x\right)+F_{2}\left(y\right)=\sum_{j=0}^{49}A_{j}\left(\cos\;jx+\cos\;jy\right)$$

Considering that $C_{mn}$ is a symmetric matrix, we obtain $C_{0,n}=C_{m,0}$ for *m* = *n*. Therefore, the final expression for the out-of-plane deflections can be written as

(18) $$\begin{array}{c} w\left(x,y\right)\approx \frac{1}{4}x^{2}y^{2}C_{0,0}-\frac{1}{2}\sum_{m=1}^{18}\frac{C_{m,0}}{m^{2}}\left(x^{2}\cos\;my+y^{2}\cos\;mx\right)\\ +\sum_{m=1}^{18}\sum_{n=1}^{18}\frac{C_{mn}}{m^{2}n^{2}}\cos\;mx\cos\;ny+\sum_{j=0}^{49}A_{j}\left(\cos\;jx+\cos\;jy\right) \end{array}$$

The various $A_{j}$ and $C_{mn}$ coefficients for the present case are presented in Appendix C.

**Figure 4:**
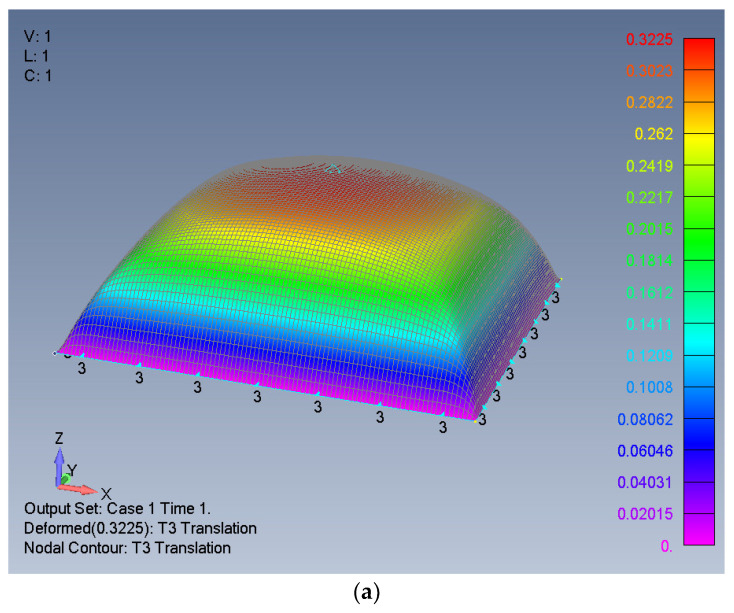

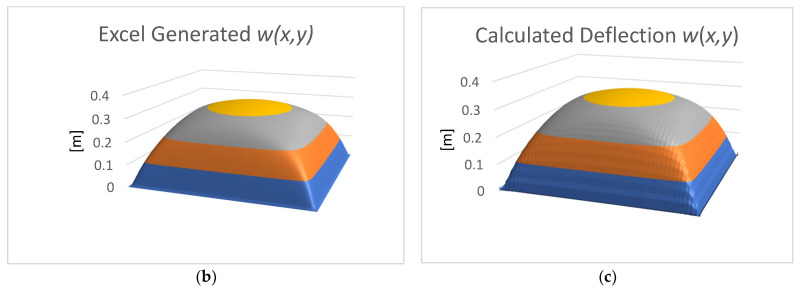
The distribution of the plate’s lateral deflection: (**a**) FEA output, (**b**) Excel-generated deflection, (**c**) Equation (18)-calculated deflection.

The calculated deflection according to Equation (18) is shown graphically in Figure 4c and is shown to be practically identical to the FEA output and the Excel-generated deflection, except minor numerical ripples on the plate’s surface, as presented in Figure 4a,b.

Note that the calculated plate deflection at the mid-point x=y=0 using Equation (18) is calculated to be:

(19) $$w\left(0,0\right)=w_{0}=\sum_{m=1}^{18}\sum_{n=1}^{18}\frac{C_{mn}}{m^{2}n^{2}}+\sum_{j=0}^{49}2A_{j}=0.417727-0.09517=0.32256[m]$$

while the FEA mid-point deflection is found to be 0.32244 [m] and the Excel mid-point deflection is 0.32250 [m]—without doubt an excellent agreement.

**Figure 5:**
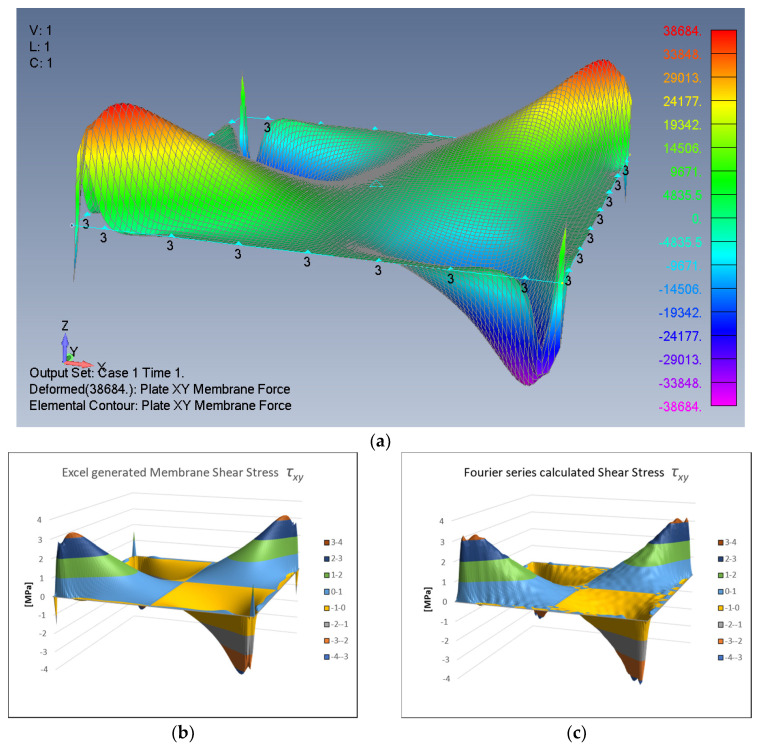
Shear stress distribution: (**a**) FEA output, (**b**) Excel stress (max value ±3.2237 MPa), (**c**) Equation (20)-calculated stress.

Next, the shear stresses expressed by the Fourier series are derived. Being an antisymmetric function about the x,y axes (see Figure 5a), the shear stress $\tau_{xy}\left(x,y\right)$ can be approximated by a double summation sine-sine Fourier series, namely

(20) $$\tau_{xy}\left(x,y\right)\approx \sum_{m=1}^{19}\sum_{n=0}^{19}S_{mn}\sin mx\sin ny$$

The symmetry of the stress function shape about the main diagonals causes the $S_{mn}$ coefficients matrix to be a symmetric matrix. The matrix $S_{mn}$ is calculated and presented in Appendix C.

The shear stress map is then calculated and is depicted in Figure 5c, which is practically identical to Figure 5a,b, calculated by the FEA and the Excel spreadsheet, respectively. Obviously, a higher number of coefficients would create a better similarity.

Note that the in-plane shear stress level is represented by the vertical *z* axis, where the positive and negative values indicate the shear direction as defined by the FE software (Femap v2021.1). Note also that the shear stress is close to zero at the edges and at the *x*, *y* axes themselves, as expected.

Finally, the mathematical expression for the ASF is derived, using the following expression (based on Equation (8))

(21) $$\tau_{xy}\left(x,y\right)=-\frac{\partial^{2}\varphi }{\partial x\partial y}$$

Twice integrating Equation (21) with respect to *x* and *y* yields the mathematical expression for the ASF:

(22) $$\varphi \left(x,y\right)=-\sum_{m=1}^{19}\sum_{n=1}^{19}S_{mn}\frac{1}{mn}\cos\;mx\cos\;ny+X\left(x\right)+Y\left(y\right)$$

where $S_{mn}$ is the symmetric coefficients matrix, previously found from FEA data, and $X\left(x\right)$ and $Y\left(y\right)$ are the arbitrary integration functions to be determined. A practical way to find these integration functions is to write expressions for $\sigma_{xx}$ based on Equations (8) and (22) and then compare them with the numerical results of the FEA. These yield

(23) $$\sigma_{xx}=\frac{\partial^{2}\varphi }{\partial y^{2}}=\sum_{m=1}^{19}\sum_{n=1}^{19}S_{mn}\frac{n}{m}\cos mx\cos ny+Y\prime \prime \left(y\right)$$

**Figure 6:**
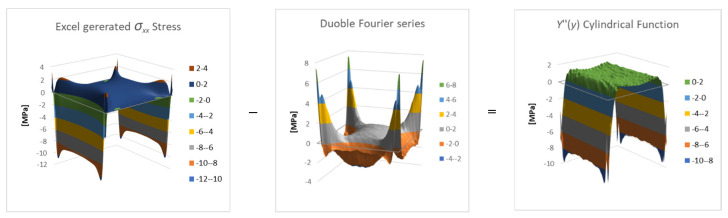
The calculated distribution of the cylindrical function *Y*″(*y*).

Depicting the *x* stresses, $\sigma_{xx}$, distribution from the FEA results (see Hakim & Abramovich [29]), calculating the double Fourier series presented in Equation (23) with the already-determined coefficients $S_{mn}$ yields a difference between the two distributions, as presented by Figure 6.

$$\sigma_{xx}\;\mathrm{Stress}-\mathrm{Double}\;\mathrm{Fourier}\;\mathrm{Series}=\mathrm{Cylindrical}\;\mathrm{function}\;Y\prime \prime \;[\mathrm{MPa}]$$

The graphs presented in Figure 6 are the results of subtraction of two known numerical functions, based on Equation (23), to obtain the numerical function of $Y\prime \prime \left(y\right)$.

The general cylindrical shape of the difference function $Y\prime \prime \left(y\right)$ suggests that it is a function of *y* only, as expected. Since this function is known numerically, it is possible to approximate it as a single Fourier series, for which a finite number of coefficients can be found, yielding

(24) $$Y\prime \prime \left(y\right)=\sum_{k=1}^{19}B_{k}\cos ky$$

A similar analysis for the function $X\left(x\right)$ leads to another series with the same number of coefficients, namely

(25) $$X\prime \prime \left(x\right)=\sum_{j=1}^{19}B_{j}\cos jx$$

The calculated numerical values of $B_{j}$ or $B_{k}$ are given in Appendix C, where $B_{j}=B_{k}\;\mathrm{for}\;j=k$.

Integrating twice Equations (24) and (25) yields expressions for $X\left(x\right)$ and $Y\left(y\right)$, written as

(26) $$X\left(x\right)=-\sum_{j=1}^{19}\frac{B_{j}}{j^{2}}\cos\;jx+a_{1}x+const.$$

(27) $$Y\left(y\right)=-\sum_{k=1}^{19}\frac{B_{k}}{k^{2}}\cos\;ky+b_{1}y+const.$$

Since both $X\left(x\right)$ and $Y\left(y\right)$ must be symmetric functions, the assumption that $a_{1}=b_{1}=0$ is found to be valid.

Then, the final expression of the ASF in [MN] units can be obtained using Equation (22) in combination with Equations (26) and (27), yielding

(28) $$\varphi \left(x,y\right)=-\sum_{m=1}^{19}\sum_{n=1}^{19}S_{mn}\frac{1}{mn}\cos\;mx\cos\;ny-\sum_{j=1}^{19}\frac{B_{j}}{j^{2}}\left(\cos\;jx\;+\;\cos\;jy\right)\;+\;Const.$$

where $S_{mn}$ and $B_{j}$ are a finite number of known Fourier coefficients given in Appendix C.

One should note that the general expression for the Airy stress function (ASF) is a novel finding, never presented in the literature.

**Figure 7:**
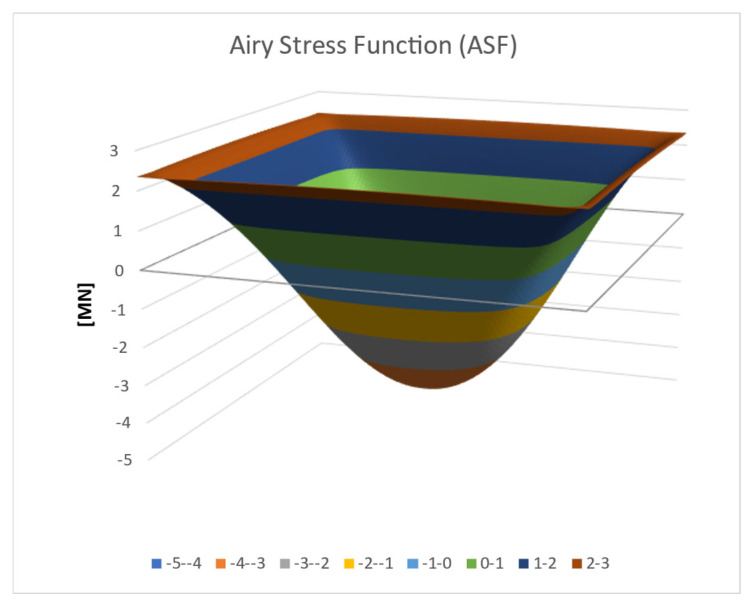
Three-dimensional (3D) ASF distribution.

The coefficients $S_{mn}$ and $B_{j}$ can now be used to calculate the approximated values of the ASF function for every $\left(x,y\right)$ point, as shown in Figure 7. One can change the vertical position by an arbitrary constant. For *Const*. = 0, the mid-point value is −4.067 [MN] and the edge value is 2.374 [MN].

The various colors in Figure 7 represent the function values in [MN] according to the legend at the bottom of the figure.

To validate the above performed calculations, the tensile stresses obtained from the ASF are recalculated and compared to other available results. From Equations (8) and (28), we obtain

(29) $$\sigma_{xx}=\frac{\partial^{2}\varphi }{\partial y^{2}}=\sum_{m=1}^{19}\sum_{n=1}^{19}S_{mn}\frac{n}{m}\cos\;mx\cos\;ny+\sum_{j=1}^{19}B_{j}\cos\;jy$$

**Figure 8:**
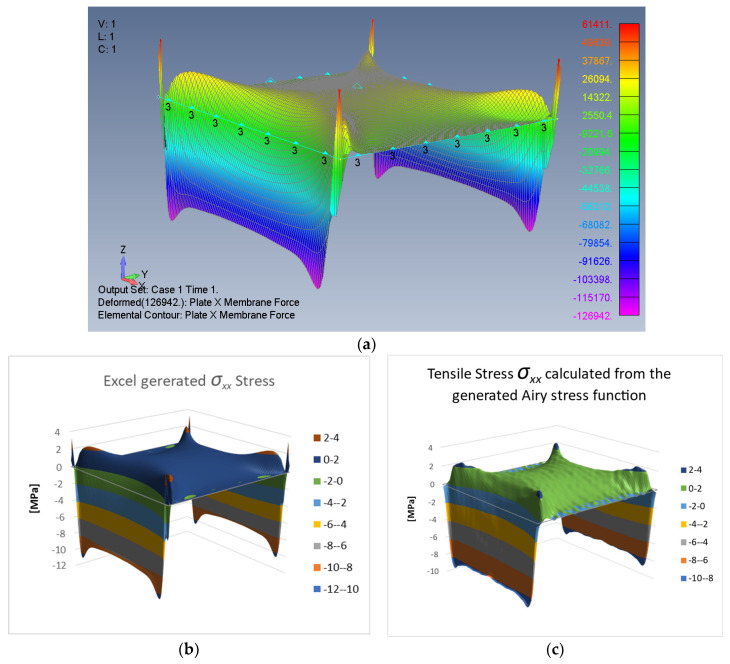
Tensile stresses *σ_xx_* (mid-point value 1.6839 MPa): (**a**) FEA output, (**b**) Excel-generated stress, (**c**) Equation (29)-calculated stress.

Calculating Equation (29) with the known coefficients $S_{mn}$ and $B_{j}$, we obtain the tensile stress distribution as given in Figure 8c.

It is clear that the calculated surface shape is generally similar to the results of the FEA shown in Figure 8a and the Excel-calculated stresses (Figure 8b), and by that the process was validated.

Note that the ripples on the surface presented in Figure 8c were due to the limited number of coefficients used in the present Fourier series.

### 3.2. Validation of the von Kármán’s Equations Set

Having numerical values for the partial derivatives of the deflection and the ASF allows us to check that the various found expressions satisfy the von Kármán equations, Equations (6) and (7). This may support the validity of the presented approximated expressions.

Equations (6) and (7) are modified to be written in residuals form by moving all terms to the left side, yielding:

(30) $$\frac{\partial^{4}\varphi }{\partial x^{4}}+2\frac{\partial^{4}\varphi }{\partial x^{2}\partial y^{2}}+\frac{\partial^{4}\varphi }{\partial y^{4}}-E\left({\left(\frac{\partial^{2}w}{\partial x\partial y}\right)}^{2}-\frac{\partial^{2}w}{\partial x^{2}}\frac{\partial^{2}w}{\partial y^{2}}\right)=0$$

(31) $$D\left(\frac{\partial^{4}w}{\partial x^{4}}+2\frac{\partial^{4}w}{\partial x^{2}\partial y^{2}}+\frac{\partial^{4}w}{\partial y^{4}}\right)-q-h\left(\frac{\partial^{2}\varphi }{\partial y^{2}}\frac{\partial^{2}w}{\partial x^{2}}+\frac{\partial^{2}\varphi }{\partial x^{2}}\frac{\partial^{2}w}{\partial y^{2}}-2\frac{\partial^{2}\varphi }{\partial x\partial y}\frac{\partial^{2}w}{\partial x\partial y}\right)=0$$

An Excel spreadsheet with the various partial derivative data was used to calculate the stand-alone derivatives and the multiplications above. Each term is a 103 × 103 numeric table representing its values at all the plate’s points. Since the equations should hold everywhere on the plate, each point is calculated separately for the equations’ value. A non-zero value indicates the deviation from von Kármán’s theory. The equations’ value matrices are then displayed graphically with some statistics and conclusions.

**Figure 9:**
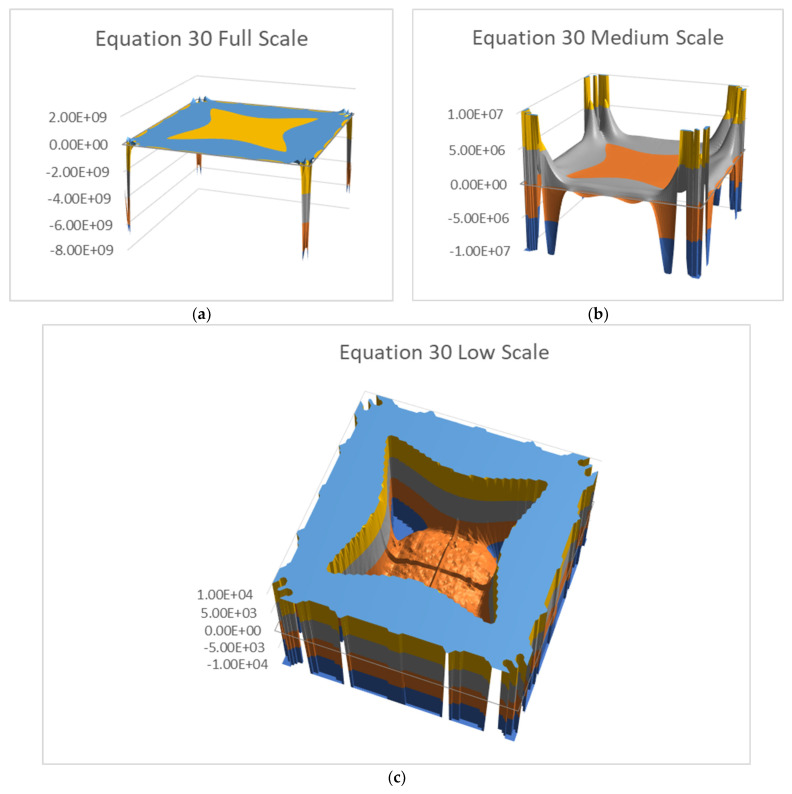
Equation (30) values with three truncation levels: (**a**) full scale, (**b**) ±1 × 10^7^ truncation, (**c**) ±1 × 10^4^ truncation.

**Figure 10:**
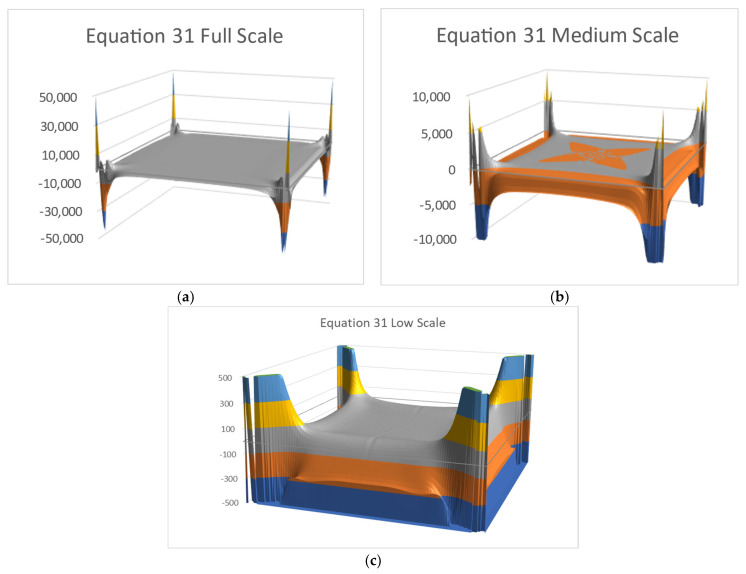
Equation (31) values with three truncation levels: (**a**) full scale, (**b**) ±1 × 10^4^ truncation, (**c**) ±500 truncation.

Figure 9 and Figure 10 show Equations (30) and (31) for three truncation levels. The three truncations are necessary in order to see the function shape properly. Using a single image would hide most of the graphical information. The full scale (a) shows the corners well, but the middle area is flat. The second truncation (b) shows more details, while (c) shows the middle area real shape. Note the vertical axis scale differences.

**Figure 11:**
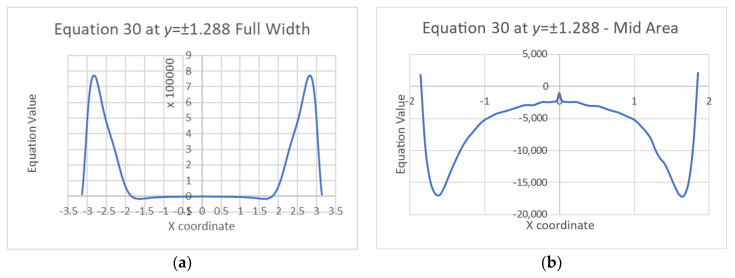
Equation (30) at an arbitrary cross-section *y* = ±1.288: (**a**) entire plate, (**b**) magnification of the middle area.

**Figure 12:**
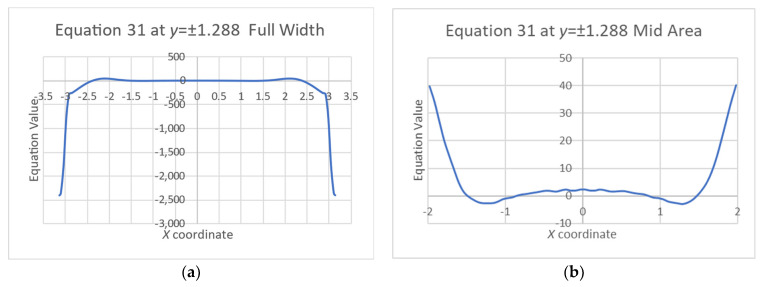
Equation (31) at an arbitrary cross-section *y* = ±1.288: (**a**) entire plate, (**b**) magnification of the middle area.

To better visualize the variation of the equations’ values across the plate, cross-section graphs are shown in Figure 11 and Figure 12.

Obviously, the sharp changes of the stress functions near the edges, and even more near the corners, create very high derivative values that do not zero the equations. Nevertheless, the plate’s middle zone has better results. Table 1 shows the average values and the standard deviations (Std) of both Equations (30) and (31) at a 3.14 × 3.14 m square in the middle zone of the plate, i.e., 25% of the plate area.

In Table 1, the first column, Equation (30), shows the average calculated value of the left side of Equation (30) and its standard deviation (Std). A value close to zero would indicate that the calculated data agree well with that equation. The second column, Equation (31), is similar but for the other equation.

From these results, one can conclude that the suggested numerical model conforms well with the second von Kármán equation, Equation (7), written in residual form as Equation (31), at the middle zone of the plate. However, for the first von Karman equation, Equation (6) (in residual form, Equation (30)), the proposed numerical model yields a relatively high error, probably due to the process involved in generating the expressions for the lateral deflection and the ASF. A more refined model is expected to yield better results.

In addition, since both functions *w* and *φ* are also function of the load, their derivatives are also functions of the load. However, in the presented calculation, the load is taken as a constant. It might be possible that recalculating while considering these load functions may yield results closer to zero and thus completely validate the von Kármán equations set.

### 3.3. The Load Influence on the Lateral Deflection and Stresses

**Figure 13:**
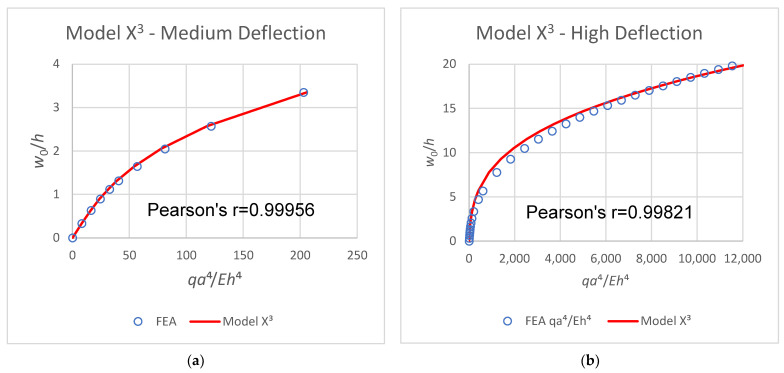
Medium deflection (**a**) and high deflection (**b**) load–deflection curves.

The common way to predict the mid-point deflection due to an applied load is to use the third power polynomial model presented by Equation (13), (see Appendix B in Hakim & Abramovich [30] for more details). For relatively medium deflections (up to four times the thickness), it works well, although considerable variability exists between various sources published in the literature (see Appendix B in [30]). For higher deflections, however, this model deviates from the actual load–deflection FEA data, as shown in Figure 13b. Note the correlation coefficients Pearson’s *r* that show the increased deviation. In these figures, both the deflection and the load are presented as dimensionless variables to make the findings more general: $\frac{w_{0}}{h}$, $\frac{qa^{4}}{Eh^{4}}$, where $w_{0}$ is the mid-point deflection, *h* is the thickness, *q* is the load, *a* is the length–width, and *E* is the modulus.

An attempt to find a better model, considering the Poisson’s ratio *ν*, yields the following inverse non-dimensional deflection–load function relation:

(32) $$\frac{w_{0}}{h}=\frac{k_{1}}{1+0.212044\left(0.09-\nu^{2}\right)}\left[k_{2}{\left(\frac{qa^{4}}{Eh^{4}}\right)}^{k_{3}}-{\left(\frac{qa^{4}}{Eh^{4}}\right)}^{k_{4}}\right]$$

where *k*_1_ = −2.1528, *k*_2_ = 1.10798, *k*_3_ = 0.22488, and *k*_4_ = 0.3101 (see Appendix C for more digits). The deflection is linearly corrected around ν=0.3.

The term “inverse” relates to the deflection being a function of the load, which is opposite to the original third order polynomial.

**Figure 14:**
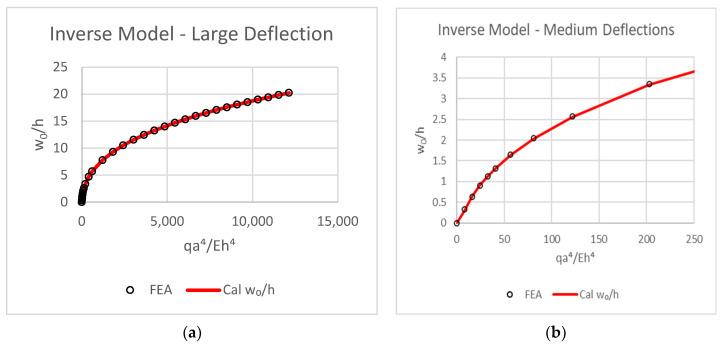
Large deflection (**a**), medium deflection (**b**).

The model presented by Equation (32) has an excellent correlation coefficient of Pearson’s *r* = 0.99999904 with the FEA data. The graphic result is displayed in Figure 14.

Note that since the function presented in Equation (32) becomes negative for $\left(\frac{qa^{4}}{Eh^{4}}\right)<3.33$, it is not valid for that region. Therefore, small deflections classical plate theory (CPT) described in Hakim & Abramovich [30] Appendix A can be used for $\left(\frac{w_{0}}{h}\right)<0.5$ or for $\left(\frac{qa^{4}}{Eh^{4}}\right)<10$ in which

(33) $$\frac{qa^{4}\left(1-\nu^{2}\right)}{Eh^{4}}=\frac{246.16}{12}\left(\frac{w_{0}}{h}\right)$$

Using the assumption presented in Equation (11), the deflection function is a multiplication of a normalized shape function and a load function. Both functions have already been found. Dividing the deflection expression Equation (18) by the mid-point deflection Equation (19) yields the normalized shape function ${SF1}_{d}$, for −π≤x≤π,−π≤y≤π and the mid-point value is 1. Hence, the multiplication of Equation (32) by the normalized shape function ${SF1}_{d}$ yields the full non-dimensional deflection function, namely

(34) $$\begin{array}{c} \frac{w}{h}\left(w,y,q\right)\approx \frac{k_{1}}{1+0.212044\left(0.09-\nu^{2}\right)}\left[k_{2}{\left(\frac{qa^{4}}{Eh^{4}}\right)}^{k_{3}}-{\left(\frac{qa^{4}}{Eh^{4}}\right)}^{k_{4}}\right]\cdot \\ \cdot \frac{1}{0.32256}\left[\frac{1}{4}x^{2}y^{2}C_{0,0}-\frac{1}{2}\sum_{m=1}^{18}\frac{C_{m,0}}{m^{2}}\left(x^{2}\cos my+y^{2}\cos mx\right)\right.\\ \left.+\sum_{m=1}^{18}\sum_{n=1}^{18}\frac{C_{m,n}}{m^{2}n^{2}}\cos mx\cos ny+\sum_{j=0}^{49}A_{j}\left(\cos jx\cos jy\right)\right] \end{array}$$

where all the coefficients are given in Appendix C and the small load case is considered in Equation (33).

The influence of the load level on the generated stresses is next investigated in order to find the Load Function ${LF2}_{t}\left(q\right)$ defined after Equation (12).

**Figure 15:**
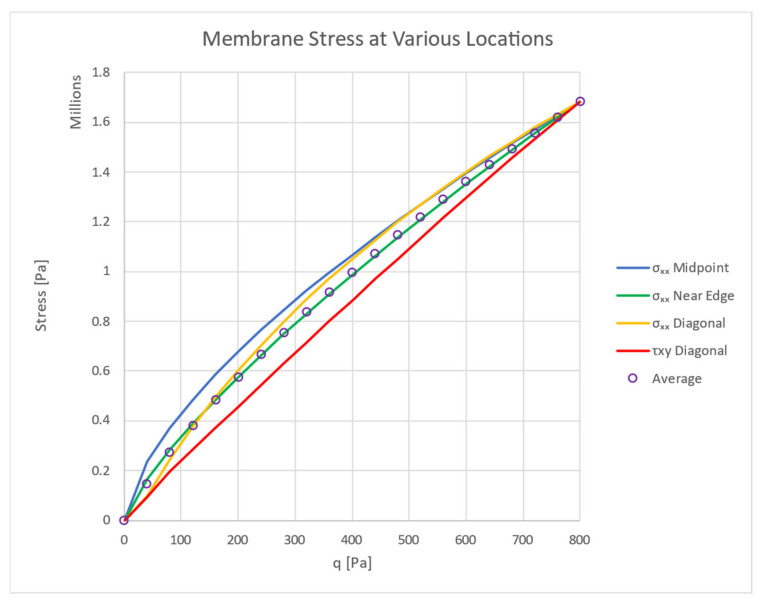
The tensile stresses at various plate points (max value 1.6839 MPa).

Using the FEA results, the stress at several indicative points are checked vs. varying loads. All stress values at the various locations are factored such that the maximum value at each location is equal to the maximum value of the tensile $\sigma_{x}^{m}$ stress at the mid-point. The factored stress vs. load curves are shown in Figure 15, where the legend lists the points’ locations.

Although differences between the various points do exist, the general shape of the lines is rather similar. In order to obtain a single stress function, we use the average of the values of these points for the calculations. Both the stress and the load are modified to be non-dimensional according to Timoshenko [31] (p. 423), in which the graph axes are:

(35) $$\frac{\sigma_{x}^{m}a^{2}\left(1-\nu^{2}\right)}{Eh^{2}}\;vs.\;\frac{qa^{4}}{Dh}=\frac{qa^{4}\cdot 12\left(1-\nu^{2}\right)}{Eh^{4}}$$

This yields a general non-dimensional expression for stresses, enabling its use for various materials and dimensions.

Using the best fitting regression of these average FEA stress data to an empirical formula yields the following stress–load expression, with an excellent correlation coefficient of *r* = 0.999995.

(36) $$\frac{\sigma_{x}^{m}a^{2}\left(1-\nu^{2}\right)}{Eh^{2}}\approx 0.054603{\left(\frac{qa^{4}\left(1-\nu^{2}\right)}{Eh^{4}}\right)}^{0.85396}-0.0050161\left(\frac{qa^{4}\left(1-\nu^{2}\right)}{Eh^{4}}\right)$$

**Figure 16:**
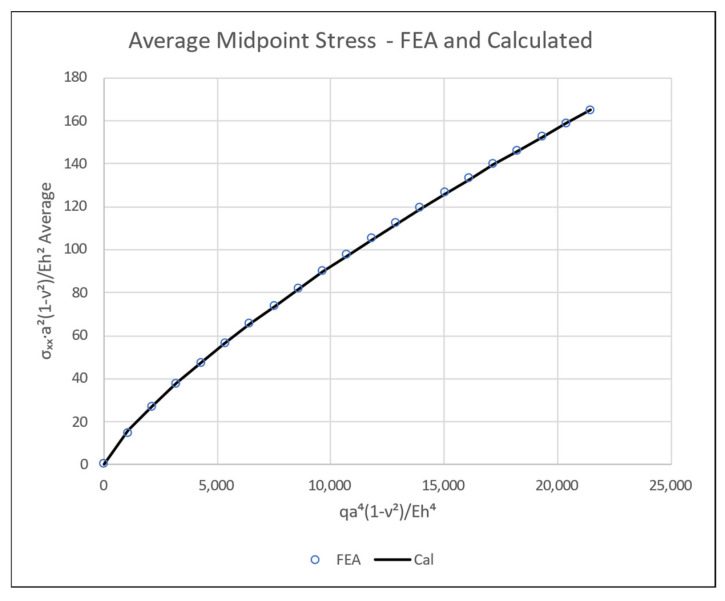
Non-dimensional mid-point tensile stress vs. non-dimensional applied pressure.

The graph of the non-dimensional mid-point tensile stress against the non-dimensional load for both FEA data and the formula calculated values is displayed in Figure 16.

### 3.4. Closed-Form Non-dimensional Expressions for Tensile and Shear Stresses

The approximated mid-point stress–load function presented in Equation (36) can now be used to find the various membrane stresses on the entire plate.

According to the assumption presented in Equation (12), the ASF is a multiplication of a normalized shape function and a load function. From this, it follows that the membrane shear and tensile stresses can also be represented with a similar multiplication. To do that, we use a shear shape function named $SF3_{\tau }$ and a tensile shape function named $SF4_{\sigma }$. The necessary involved functions have already been found before in Equations (20) and (29).

For shear stresses, dividing Equation (20) by the maximum shear stress, 3.2237 MPa (see Figure 5), yields the normalized shape function $SF3_{\tau }$ in which −π≤x≤π,−π≤y≤π and its maximum value is 1.

(37) $$SF3_{\tau }\left(x,y\right)=\frac{1}{3.2237}\sum_{m=1}^{19}\sum_{n=1}^{19}S_{mn}\sin\;mx\sin\;ny$$

Then, Equation (36), which is the mid-point non-dimensional tensile stress vs. load function, is used. However, since we need here the shear stress, we must multiply Equation (36) by the ratio of the maximum $\tau_{xy}$ = 3.2237 MPa to mid-point $\sigma_{x}$ = 1.6839 MPa.

This yields the final membrane shear stress $\tau_{xy}^{m}$ in a non-dimensional form

(38) $$\begin{array}{c} \frac{\tau_{xy}^{m}a^{2}\left(1\;-\;\nu^{2}\right)}{Eh^{2}}\left(x,y,q\right)=\\ =\frac{3.2237}{1.6839}\left[0.054603{\left(\frac{qa^{4}\left(1\;-\;\nu^{2}\right)}{Eh^{4}}\right)}^{0.85396}-0.0050161\left(\frac{qa^{4}\left(1\;-\;\nu^{2}\right)}{Eh^{4}}\right)\right]\\ \cdot \left[\frac{1}{3.2237}\sum_{m=1}^{19}\sum_{n=1}^{19}S_{mn}\sin mx\sin ny\right]\\ =\frac{1}{1.6839}\left[0.054603{\left(\frac{qa^{4}\left(1\;-\;\nu^{2}\right)}{Eh^{4}}\right)}^{0.85396}-0.0050161\left(\frac{qa^{4}\left(1\;-\;\nu^{2}\right)}{Eh^{4}}\right)\right]\\ \cdot \left[\sum_{m=1}^{19}\sum_{n=1}^{19}S_{mn}\sin mx\sin ny\right] \end{array}$$

A similar procedure is applied for tensile stresses. Dividing Equation (29) by the mid-point stress, 1.6839 MPa, yields the normalized shape function ${SF4}_{\sigma }$ in which π≤x≤π,−π≤y≤π and the mid-point value is 1, having the following form

(39) $${SF4}_{\sigma }\left(x,y\right)=\frac{1}{1.6839}\left[\sum_{m=1}^{19}\sum_{n=1}^{19}S_{mn}\frac{n}{m}\cos mx\cos ny+\sum_{k=1}^{19}B_{k}\cos ky\right]$$

Then, Equation (36) is multiplied by Equation (39) to yield the non-dimensional membrane tensile stress $\sigma_{x}^{m}$, namely

(40) $$\begin{array}{c} \frac{\sigma_{x}^{m}a^{2}\left(1\;-\;\nu^{2}\right)}{Eh^{2}}\left(x,y,q\right)=\\ =\left[0.054603{\left(\frac{qa^{4}\left(1\;-\;\nu^{2}\right)}{Eh^{4}}\right)}^{0.85396}\;-\;0.0050161\left(\frac{qa^{4}\left(1\;-\;\nu^{2}\right)}{Eh^{4}}\right)\right]\cdot \\ \cdot \frac{1}{1.6839}\left[\sum_{m=1}^{19}\sum_{n=1}^{19}S_{mn}\frac{n}{m}\cos mx\cos ny+\sum_{k=1}^{19}B_{k}\cos ky\right] \end{array}$$

where all coefficients are given in Appendix C. The other direction stress $\sigma_{y}^{m}$ can be calculated by simply switching between *x* and *y* axes.

## 4. Discussion

The present study can be considered to be significant and innovative, as, in addition to displaying numerical solutions for the plate’s in-plane stresses and deflections, it presents high-fidelity non-dimensional mathematical expressions that can be used to calculate deflections and stresses for various materials and plate dimensions. Consequentially, these expressions allow us to calculate and display for the first time the Airy stress function (ASF) and also to check the correctness of von Kármán’s equations set.

Another innovative finding is that the commonly used third power polynomial expression for a plate’s large deflection is not accurate enough for higher deflections. An improved expression is suggested for deflections up to 20 times the plate’s thickness.

The present study deals with square plates. From Hakim & Abramovich [29], it is obvious that rectangular plates with higher aspect ratios (length/width), are not a simple “stretching” of the square plate. Therefore, rectangular plates should be considered separately, following the process presented in the present study.

## 5. Conclusions

In view of what has been presented above the following conclusions can be drawn:

- Fourier series approximations produced high-fidelity closed-form expressions for the deflections and stresses on the entire plate domain.
- For the first time, a closed-form equation was derived for the Airy stress function, presented in the von Kármán equations set.
- Mathematical expressions for the load influence on the deflections and the stresses were also derived.
- The mathematical expressions were given in a non-dimensional form, enabling the use of any elastic material and plate dimensions.
- Strong compressive stresses do exist near the edges in the direction parallel to the edges. This might raise the possibility of plate local buckling there. In addition, high tensile stresses were detected, which might cause failure of the plate.
- The numerical model upholds the second von Kármán equation, Equation (7), in the plate’s middle zone, while the first equation, Equation (6), has not been fully verified by the present model.
- It was found that the third power model can describe the mid-point load–deflection relation accurately enough for moderate deflections only, up to five times the thickness. For higher-type deflections, up to 20 times the thickness, an improved (*r*≈1) model is presented and discussed.

## Figures and Tables

**Table 1:** Equations (30) and (31) statistics values.

	Equation (30)	Equation (31)
Average	−3183.38	0.7605
Std	3740.91	2.3635

## Data Availability

Not applicable.

## Appendix A. Finite Difference Schemes Used in the Present Analysis

**First Derivatives:**

Forward difference: $f\prime (x)\approx \frac{f\left(x+h\right)-f(x)}{h}$
Central difference: $f\prime (x)\approx \frac{f\left(x+h\right)-f(x-h)}{2h}$
Backward difference: $f\prime (x)\approx \frac{f\left(x\right)-f(x-h)}{h}$

**Second Derivatives:**

Forward difference: $f\prime \prime (x)\approx \frac{f\left(x+2h\right)-2f\left(x+h\right)+f(x)}{h^{2}}$
Central difference: $f\prime \prime (x)\approx \frac{f\left(x+h\right)-2f\left(x\right)+f(x-h)}{h^{2}}$
Backward difference: $f\prime \prime (x)\approx \frac{f\left(x\right)-2f\left(x-h\right)+f(x-2h)}{h^{2}}$

## Appendix B. Remarks on Fourier Series Approximation and Finding the Best Fit Coefficients

In order to handle the deflection and the stress functions analytically, the approximate Fourier series that are close enough to these functions were defined and used.

For deflections, this process was done on one of the deflection fourth derivatives. The reason for taking the highest derivative degree was that deriving a finite Fourier series may increase the numerical “noise”, while integration reduces it. By taking the highest possible derivative, it was necessary only to integrate, gaining less numerical noise. For deflections, the fourth mixed derivative $\frac{\partial^{4}w}{\partial x^{2}\partial y^{2}}$ was used. For stresses, however, the shear stress was used (not its derivative) since the numerical derivations were too “noisy” for this approximation. For deflection, the derivative symmetry suggested the use of a double summation cosine-cosine series, while for the shear stresses, the anti-symmetry suggested a double summation sine-sine series.

Since the analytic functions were unknown, it was not possible to use a closed-form formula to yield the series coefficients. However, as 103 × 103 data points of the functions were already known, it was possible to find a finite number of coefficients that make the above double summation best fit the data. This was done through Excel’s Solver by minimizing the sum of squares of the difference between the calculated and the actual data—a least squares regression analysis. Solver used internally a generalized reduced gradient (GRG) non-linear solving method. Solver has a limit of 200 variable cells that can be looked for, limiting the coefficients matrix size to be 14 × 14 = 196. Nevertheless, since the functions were symmetric and anti-symmetric, their coefficients matrices were symmetric. This reduced the number of independent coefficients to be not N × N, but 0.5 × N × (N + 1), which is a lower number. Therefore, using Solver, we could find 190 variables that, with the matrix symmetry, yielded 361 (19 × 19) coefficients. Due to the large number of variable cells and the matrix size, it took Solver code several hours to find the convergent solution.

## Appendix D. Large Deflection Literature Survey

This section is based on a previous paper of the authors Hakim, G.; Abramovich, H. [29], where various sources about the large deflection problem have been reviewed. This review is repeated here to enhance the view of this vast problem.

Browsing the literature reveals that the non-linear behavior of flat plates using von Kármán’s two equations was mainly investigated for the transverse deflections of the plate, with the in-plane (the membrane)-generated stresses being less discussed. NACA had allocated a lot of efforts to investigate the issue by publishing technical reports in the years 1941–1951 (see typical reports in [2,3,4,5,6,7,8,9]). Various methods were employed, such as multiplying double Fourier series results in quadruple series with a large number of terms, leading to numerical tables and graphs with the membrane stresses in the *x* and *y* directions being calculated at the mid-point of a square plate and mid-point edges, and the shear stresses set to zero in Levy [3]. The numerical work was further enhanced in an experimental study on aluminum-squared plates [4]. Another interesting study is presented in Levy [5] for a clamped plate having an aspect ratio of 1.5 and undergoing large deflections. Their results differed only by 3% from an infinitely long plate, thus implying that long plates should be treated as infinitely long and the in-plane stress distribution along lines parallel to the edges going through the plate center do not change significantly.

Following the studies presented by Samuel Levy [3,4,5,6], Wang [7,8] solved the large deflection problem by employing two finite differences schemes, the successive approximations and relaxation method to yield a good comparison with Levy’s results. He considered an all-around immovable clamped plate and all-around simply supported movable boundary conditions. Both square and rectangular plates were presented with the in-plane stresses being calculated at three points, center of the plate, long edge mid-point and short edge mid-point, without indicating the presence of compression-type stress. One should note also the study presented in Yen [9] for sandwich-type plates, for which numerical and experimental results were presented and well compared.

In 1954, Berger [10] assumed that the strain energy due to the second invariant of the middle surface strain can be neglected, leading to a solution of the von Kármán equations set. This neglection would mean that for the large deflections case, the plate’s bending resistance is low, and the plate would behave as a pure membrane. The study presented results for circular and rectangular flat plates for both clamped and simply supported boundary conditions, with deflections and stresses being presented graphically and numerically at certain points on the plate.

In 1969–1970, Scholes and Bernstein [11] and Scholes [12] presented approximate large deflections solutions using energy methods for all-around simply supported rectangular plates [11] and all-around clamped plates [12]. A good comparison with experimental results was reported in Scholes and Bernstein [11]. They employed Timoshenko’s [31] mentioned idea to divide the loading path into a first part, which would cause bending, and a secondary part leading to membrane stretching, and the load–deflection curve was calculated by a finite differences scheme. The clamped case being dealt with in Scholes [12] presents stresses and deflections calculations and comparison with measured results. Maximal values are presented for a pressure-loaded plate to enable efficient design.

Li-Zhou and Shu [13] used the perturbation variational method to solve the large deflections problem of rectangular plates under transverse pressure, leading to an analytical expression for displacements and stresses. They reported a good comparison with available experiments.

Bert et al. [14] also addressed von Kármán’s equations for orthotropic rectangular plates. The solution was obtained and presented using the differential quadrature method. The boundary condition used in their study was all-around simply supported and all-around clamped, both immovable. They reported deflections, membrane, and bending stresses are in good agreement with known solutions. Values of the stresses were calculated at the plate’s mid-point as a function of the applied transverse load. Yeh and Liu [15] also addressed the issue of the approximated analytic solution for the orthotropic von Kármán equations. The presented solution led to an expression for the self-mode frequency. Numerical and graphical solutions were presented only for deflections, while the stress distribution was not dealt with in the study.

More recent studies, such as by Wang and El-Sheikh [16], presented results for the von Kármán equations by multiplying Fourier series, obtaining quadruple sums, and equating similar terms in the results series. The output was a non-linear algebraic equations system with 1, 3, 4, 6, or 9 equations and unknowns, according to the number of terms taken for the series. This system was solved for every desired point on the plate. For that, the authors used numerical tools based on the generalized reduced gradient (GRG) method. They also presented a closed-form solution for the mid-point deflection using the first term only having the form of q = α · *w* + γ · *w*^3^ (q = transverse load, *w* = lateral deflection, and α, γ = fitted constants). However, there are indications from other sources that the use of only one term is not accurate enough and has serious deviations from reality. An interesting solution for the Föppl–von Kármán equations set was presented by Bakker et al. [32] using an approximated analytic solution. Thanks to the simplicity of the trial function, the bending and the membrane loading influences were separated, easing the solution process. The results were compared to ANSYS FEA v2023-R1 (Engineering Simulation Software, Canonsburg, PA, USA) results with less than 10% difference. Six combinations of boundary conditions and four loading cases made the results presentation rather complex. Stresses were presented using various formulas without any graphical outcome.

Ugural [17], in his book, Ch.10, presented approximate solutions for circular thin plate S–I (simply supported, immovable edge). However, for the solution for a thin rectangular plate, he assumed membrane-only stresses (no bending resistance) at the mid-point, with SSSS–I boundary conditions.

Razdolsky [18] also presented approximate solutions for rectangular SSSS–I rectangular plates with deflections and stresses calculated for several aspect ratios. He converted the stress expressions of Levy [3,4,5,6] through the minimum potential energy method to computer executable algorithms. His square plate deflection curve was found to be between Timoshenko [31] and Levy [3,4,5,6] curves, while for the stresses, no direct comparison was presented.

Turvey and Osman [19] performed numerical analysis with finite differences dynamic relaxation (DR) of square isotropic Mindlin (shear deformable) plates. His results are said to be in “generally good agreement” with Alamy and Chapman (1969) and Rushton (1968), but no comparison was shown.

Paik et al. [20] have developed complex expressions for thin plates’ large deflection using the Galerkin method. Their example included both transverse and axial edge compression load. Since the results showed only in-plane loads on the edges, it is difficult to compare it to a case without these loads.

Nishawala [21] handled both non-linear beams and plates. For plates, both movable and immovable edges were displayed. Several other sources were compared for deflections, but without stresses. He suggested a third-degree polynomial load–deflection expression for the plate mid-point.

Jianqiao Ye [22] used both boundary elements (BE) and finite elements (FE) to calculate deflection and mid-point stress for both simply supported and clamped immovable edges. A comparison with Boshton (1970) was made, yielding a good agreement.

Abayakoon [23] studied beams as the main subject, while presenting also plates using a third-degree polynomial mid-point deflection expression. A deflection comparison was made to Timoshenko [23] and others. Stresses were simulated for ribs stiffened plates only, which cannot be compared with thin plates.

Seide [24] presented an expression for deflection, but for stresses it was limited to an infinitely long plate, which cannot be used for a square plate.

Parker [25] solved the plate problem with finite differences. He presented membrane stress results with good agreement with Levy [3] but less good with Wang [8].

Belardi et al. [26] analyzed a circular plate made of shear deformable orthotropic composite materials. While the material had Cartesian *XY* orthotropy, the other variables in the analysis were polar. The shear deformations were calculated using the FSDT (first order shear deformation theory). Deflections and rotations were presented. Stresses were considered, but without presented results.

Plaut [27], in a recent study, used Reissner theory for plates, which allows large strains (not to be confused with shear deformation) for circular and annular thin plates with both movable and immovable BC. Results for various loading cases were presented, but for deflection only. No in-plane stresses were considered.

Finally, Shufrin et al. [28] solved the problem of laminated rectangular plates under large deflections with a semi-analytic method considering the coupling coefficients tension–bending and bending–twisting. The non-linear partial differential equations were converted to an iterative process of an ordinary non-linear differential equation according to the Kantorovich method. The result was large mathematical expressions, calculated and compared to ANSYS FEA (Engineering Simulation Software, Canonsburg, PA, USA.) with good agreement. Several cases of local loads (patch type load) were also demonstrated. In-plane stresses including shear stress were partially given along certain lines and loading arrangements.
